# Geocorrection of Airborne Mid-Wave Infrared Imagery for Mapping Wildfires without GPS or IMU

**DOI:** 10.3390/s21093047

**Published:** 2021-04-27

**Authors:** Gabriela Ifimov, Tomas Naprstek, Joshua M. Johnston, Juan Pablo Arroyo-Mora, George Leblanc, Madeline D. Lee

**Affiliations:** 1 Flight Research Laboratory, Aerospace Research Centre, National Research Council Canada, 1920 Research Private, Ottawa, ON K1V 1J8, Canada; Tomas.Naprstek@nrc-cnrc.gc.ca (T.N.); JuanPablo.Arroyo-Mora@nrc-cnrc.gc.ca (J.P.A.-M.); George.Leblanc@nrc-cnrc.gc.ca (G.L.); 2 Canadian Forest Service, Great Lakes Forestry Centre, 1219 Queen St. E., Sault Ste. Marie, ON P6A 2E5, Canada; Joshua.Johnston@canada.ca; 3 Department of Geoscience and Petroleum, Faculty of Engineering, University of Science and Technology, S. P. Andersens veg 15a, 7031 Trondheim, Norway; Madeline.Lee@ntnu.no

**Keywords:** wildfire, fire monitoring, mid-wave infrared, airborne sensor, geocorrection

## Abstract

The increase in annual wildfires in many areas of the world has triggered international efforts to deploy sensors on airborne and space platforms to map these events and understand their behaviour. During the summer of 2017, an airborne flight campaign acquired mid-wave infrared imagery over active wildfires in Northern Ontario, Canada. However, it suffered multiple position-based equipment issues, thus requiring a non-standard geocorrection methodology. This study presents the approach, which utilizes a two-step semi-automatic geocorrection process that outputs image mosaics from airborne infrared video input. The first step extracts individual video frames that are combined into orthoimages using an automatic image registration method. The second step involves the georeferencing of the imagery using pseudo-ground control points to a fixed coordinate systems. The output geocorrected datasets in units of radiance can then be used to derive fire products such as fire radiative power density (FRPD). Prior to the georeferencing process, the Root Mean Square Error (RMSE) associated with the imagery was greater than 200 m. After the georeferencing process was applied, an RMSE below 30 m was reported, and the computed FRPD estimations are within expected values across the literature. As such, this alternative geocorrection methodology successfully salvages an otherwise unusable dataset and can be adapted by other researchers that do not have access to accurate positional information for airborne infrared flight campaigns over wildfires.

## 1. Introduction

As wildfire events have increased in many areas of the world with catastrophic consequences [1,2,3,4], the ability to accurately monitor these events at different spatial and temporal scales is becoming a key concern and focus for governments around the globe [5,6,7]. Active wildfire monitoring e.g., [8], rapid turn-around of data processing, and computation of fire mapping products are all fundamental to understanding the life cycle of fires, detecting spatial patterns, mitigating their potential damage, and implementing response and recovery activities. Efforts to deploy different observation platforms [8,9,10,11,12,13] is an essential task aiming to better understand, for instance, how climate change influences wildfire behaviour [14,15,16]. Remotely sensed data from infrared sensors (e.g., mid-wave infrared and long-wave infrared) are able to capture information from both naturally-occurring and controlled-fires regardless of time of day [17,18,19].

The importance of accurate detection of wildfires is that it allows the users of this technology to better address aspects such as fire behaviour [20], build fuel consumption models [21], and learn how they impact their surrounding environments [17,22]. To date, spaceborne and manned airborne platforms are commonly used to study wildfires [17,23,24], with the inclusion of unmanned aerial vehicles (UAV) into the airspace still being developed [19,25,26]. However, each platform provides unique data over top of the fire. For instance, satellite based infrared sensors provide landscape level information at a moderate (e.g., ~0.4–1.0 km) spatial resolution e.g., [8,27], however, they have very defined orbital paths and revisit periods which are often inadequate for operational purposes. Moreover, spaceborne sensors require independent data for the calibration and validation of fire satellite products [28,29]. Given the highly dynamic behaviour of wildfires [30], the use of airborne platforms for wildfire mapping [17] are ideal for near real-time characterization at operational scales (e.g., 10 km × 10 km) and fine spatial resolution (~1–5 m). Thus, airborne infrared imagery acquired over wildfires is adequate to generate end-products such as fire-front locations [30], rate of spread [24], fire radiative power and fire-line intensity [31]. These estimations can be further compared and/or integrated with satellite imagery, such as Sentinel-3 Sea and Land Surface Temperature Radiometer (SLSTR) [12], the Visible Infrared Imaging Radiometer Suite (VIIRS) [8] and the future Canadian WildFireSat [13], to establish a wildfire management system at local and national scales.

Considering airborne platforms, a key aspect of wildfire mapping is the process of geocorrection and geo-registration [9,24]. The objective of the geocorrection process is to compensate platform and/or sensor motion so that the final image is represented in a grid format and usually resampled to a specific cell size [32]. Geo-registration or georeferencing refers to the process where the goal is to align the corrected image to a local coordinate system with a given ellipsoid and datum [32]. Processing thermal infrared data to detect, for example, fires’ heat signatures and registering them to a coordinate system is ideal for most applications [17]. However, the geocorrection and registration process can be challenging within wildfire mapping, particularly at the airborne scale when Inertial Measurement Unit (IMU) and precise Global Navigation Satellite System (GNSS) data are not available [9,33]. For an effective georeferenced dataset, a user will, in general, require a quality GNSS unit capable of differential post processing [34]. As well, a high-frequency inertial measurement unit (IMU) that records the sensor’s attitude (i.e., roll, pitch, yaw) [32] is often required. While GNSS positioning is a standard feature on most survey aircraft’s navigation system, ideally the thermal sensor would also have dedicated high accuracy GNSS and IMU devices. However, this might not be the case due to on-board space limitations, or budgetary constraints [17]. As such, a geocorrection method, that can achieve high accuracy in the absence (or malfunction) of GNSS/IMU, can be valuable for maximizing the utility of the thermal imagery. While not necessarily common, equipment malfunction can occur during a field campaign, particularly in the context of wildfires where activities are carried out under both environmentally challenging and high operator stress conditions. As airborne data is expensive and labour intensive, if a malfunction in the GNSS or IMU does occur then solutions to recover the geolocation information are needed.

Here we present a novel data loss minimization methodology based upon the malfunction of both GNSS and IMU instruments in an airborne thermal infrared field campaign over an active wildfire. While specific to the issues encountered in this field campaign, the method can be easily applied to any scenario where infrared data does not have associated GPS or IMU information, whether due to equipment issues or imagery that has been acquired without the associated positional instruments. The method is a two-step semi-automatic geocorrection process that outputs image mosaics from airborne infrared video input. The first step involves taking the infrared video as individual frames and using an automatic image registration method to develop the initial mosaic. This step was followed by georeferencing the imagery using high-resolution GeoEye (1.84 m) satellite imagery acquired over the same area. While the images produced by this first step can be used for some basic, non-georeferenced analysis, the second step is highly beneficial as it aligns the orthoimage with distinct geographical features, as well as, assists in accounting for larger warping effects that occur in the imagery due to significant aircraft motion. The final output is a set of accurate orthoimages that can be compared with other GPS-referenced data and analyzed for purposes such as fire end-products (e.g., fire radiative power).

## 2. Data Collection

### 2.1. Field Campaign

**Figure 1:**
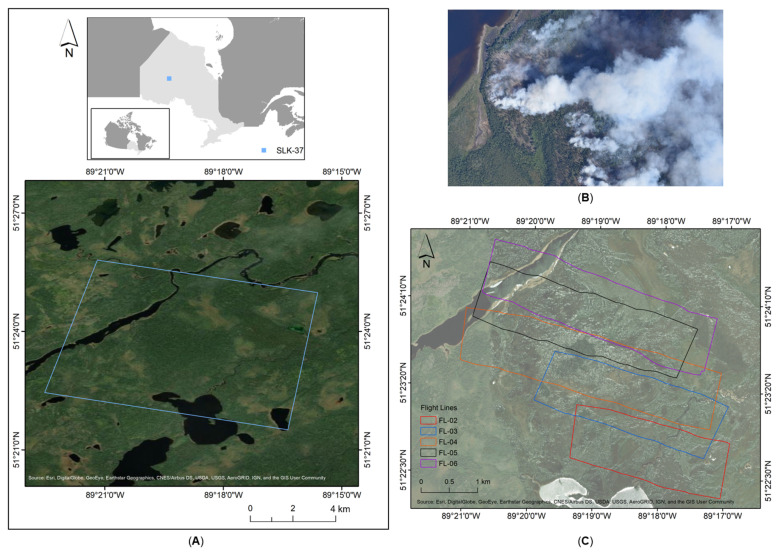
(**A**) Sioux Lookout (SLK-37) site located near Pickle Lake, Northern Ontario, Canada. FLIR MWIR data was recorded twice in successive alternating directions over eight flight lines between 2 August and 3 August 2017. (**B**) Photograph taken over the study area acquired on 3 August 2017, at 11:27 (EST). (**C**) Boundaries of the five selected flight lines (i.e., FL-02 to FL-06) for data analysis.

The airborne field campaign was a collaborative effort between the Flight Research Lab of the National Research Council Canada (NRC) and the Canadian Forestry Service of Natural Resources Canada. The two primary goals of the campaign were: (1) quantify wildland fires intensity and progression, and, (2) compare the fire’s radiative emissions of the airborne campaign to those determined from a coincidental overpass of Sentinel-3a Sea and Land Surface Temperature Radiometer (SLSTR). The project employed NRC’s Twin Otter survey aircraft that collected Forward Looking InfraRed (FLIR) mid-wave infrared (MWIR) imagery (Section 2.2) over the 37th Being Observed (BOB) wildfire in the Sioux Lookout district (SLK-37), near Pickle Lake, Northern Ontario, Canada (Figure 1A,B). 

A total of six flights (F) were conducted, however, due to weather and technical issues, only two, F-03 and F-04, produced reliable datasets. The details of these two flights are presented in Table 1. The flight patterns were flown in successive alternating directions, designed to provide overlapping data (i.e., 20–30% overlap) at the edge of each flight line. The overlap ensured total coverage of the area and allowed for the development of good quality mosaicked images. Taking into account the dynamic nature of a fire, the flight lines were planned according to the information provided by the local fire management headquarters. To cover the full extent of SLK-37, eight flight lines were preplanned and collected twice during both nighttime and daytime flight campaigns. A total of 32 flight lines (FL) were flown during both daytime (16 flight lines) and nighttime (16 flight lines) operations between 2 August and 3 August 2017. Active fire was present on five of these eight flight lines, which were selected for data analysis. The overall area (~6.5 km by 7.5 km), covered by these five flight lines (i.e., FL-02 to FL-06), is shown in Figure 1C.

### 2.2. FLIR Sensor

The thermal system used in this study was the FLIR SC8303 sensor (FLIR Systems Inc., Wilsonville, OR, USA) that covers the 3.0 µm to 5.0 µm MWIR region (Table 2). For this project, the infrared sensor was equipped with a 3.74 µm narrow bandpass flame filter. This flame filter has the same spectral band center location as the S7 and F1 channels of the Sentinel-3a SLSTR instrument [12].

The FLIR instrument can acquire data at different integration times in succession. An integration time is defined as the length of time (ms) that the sensor’s shutter is open for a single frame. Because wildfires are unique situations that have a broad array of temperatures that can range from ambient up to nearly 1000 °C [35], the integration time has a great impact on the imagery collected by the sensor. Short integration times only capture high temperature values, while long integration times are required to measure low temperature values. As such, the ability to collect multiple images at different integration times allows for a high dynamic range image and minimizes saturation by extreme values. In any digital imager the measured energy causes the detector to produce a signal voltage that is transferred to an A/D converter and results in a specific digital count related to the voltage amplitude. The FLIR sensor’s 14-bit dynamic range creates count values from 0 to 16,383 proportional to the incident energy at that integration time. These digital counts can then be transformed into temperature and radiance values via calibration relationships that are specific to the individual detector. Different integration times are selected so that the dynamic range of the signal counts can be maximised to ensure proper confidence in the measurement and visualization of the fire.

For the FLIR instrument in this work, pre-flight ground testing and laboratory measurements were performed in July 2017 prior to its installation aboard the aircraft to ensure accuracy of measurements and identify optimal integration times [36]. The full range of the blackbody source (0–9999) was captured under four integration times: 1.4 ms, 0.3 ms, 0.04 ms, and 0.0021 ms. Data was acquired using all four integration times during the 2017 flight campaign.

### 2.3. Twin Otter Survey Aircraft Hardware

While in the aircraft environment, there are three separate sensor systems used to capture the wildfire data. The first is the FLIR sensor that collects imagery at a rate of 30 Hz (i.e., 4 × 7.5 Hz given the four integration times) and is GPS time-tagged from an Inter-Range Instrumentation Group (IRIG) Time B, a generalized time measuring device for electronics, whose category “B” outputs 100 Hz data. The second system, the Databoss, records a spatial subset of the FLIR data at 30 Hz, as well as real-time aircraft system GPS information (i.e., GPS time, latitude, longitude, and heading) at 1 Hz. This system is in place for the on-board survey team to see the FLIR data as it is recorded and that the ground target area is properly acquired. This is necessary for real-time quality assurance to ensure no frames are dropped due to lack of available CPU resources, as the FLIR recording software pauses the live IR camera feed while recording. Lastly, the aircraft’s onboard Data Acquisition System (DAS) collects raw IMU data at 100 Hz and raw GPS pseudo-range data at 1 Hz. Typically, the Twin Otter raw GPS is processed using a nearby GPS base station to arrive at differential GPS data, and this is post-processed with the raw IMU data in a Kalman filter [37]. This process outputs a combined position/attitude solution at 100 Hz which is then associated with the GPS-time tagged FLIR data.

There were two separate points of failure in this process during the 2017 campaign: (1) the FLIR sensor never received proper timing information from the IRIG Time B, and (2) the aircraft’s IMU failed to record at the correct frequency. As a result, there was no GPS time to associate the FLIR imagery with the rest of the GPS information, and no high-resolution Kalman filter combined solution that could be calculated.

## 3. Methods

### 3.1. Geocorrection Process

**Figure 2:**
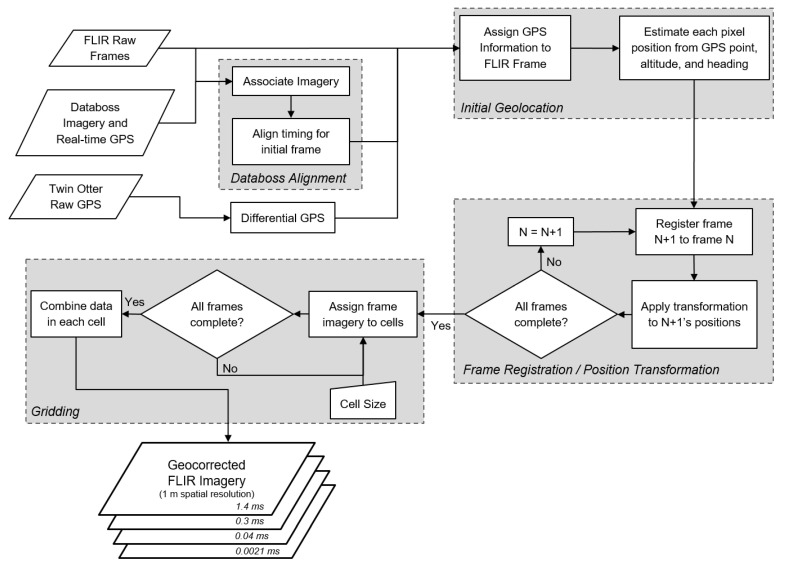
Workflow representation of the adjusted data and processing steps to compute GPS-tagged orthoimages of FLIR airborne data.

To minimize the impact of the hardware issues encountered during the 2017 field campaign, an alternative geocorrection method was developed (Figure 2). The methodology can be divided into the following steps: Databoss alignment, initial geolocation, frame registration, and gridding. This methodology was developed and performed using MATLAB Version 2016a, and the Image Processing Toolbox (The MathWorks, Inc., Natick, MA, USA). The specific code for this process can be found in [38].

#### 3.1.1. Initial Imagery Setup and Databoss Alignment

The first step in the process was to export each FLIR video frame for all integration times into separate mat files using the FLIR ResearchIR software Version 4.90.9.30 (FLIR Systems Inc.). Each integration time was exported for each flight line and processed as a complete dataset. The following step was the alignment to the Databoss GPS information. By comparing the recorded Databoss video, used as a secondary viewing system in-flight, with the raw FLIR imagery, a linear transformation from the raw FLIR frame number to the Databoss GPS could be found. This gave us access to real-time processed GPS position, altitude, and heading at 1 Hz.

#### 3.1.2. Initial Geolocation

**Figure 3:**
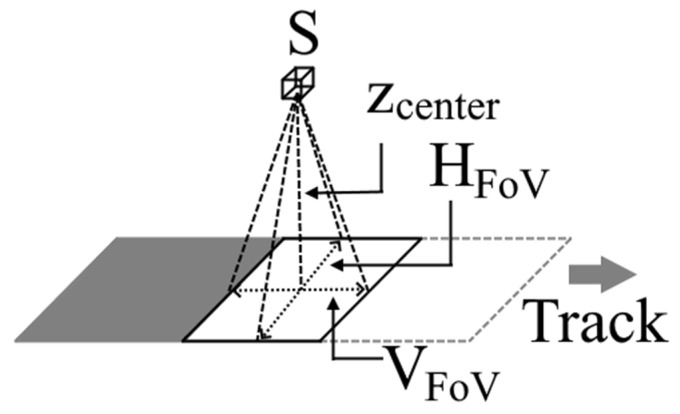
Calculation for the horizontal (H_FoV_) and vertical (V_FoV_) field of view (FoV) with respect to the sensor (S) and the aircraft-ground separation below the central pixel (Z_center_).

Using the associated 1 Hz GPS data found in the previous step, an initial geolocation could be determined. Due to lack of IMU information, these readings were assumed to be recorded during flat and level flight (i.e., the sensor is always pointed nadir). Taking into account the lever arm distance of the GPS antenna to the FLIR sensor, we could use the GPS information and FLIR lens properties to calculate the horizontal and vertical fields of view, H_FoV_ and V_FoV_, respectively:

(1) $$H_{\mathrm{FoV}}{=z}_{\mathrm{center}}\left(\frac{S_{H}}{\mathrm{EFL}}\right),$$

(2) $$V_{\mathrm{FoV}}{=z}_{\mathrm{center}}\left(\frac{S_{V}}{\mathrm{EFL}}\right),$$

where, S_H_ and S_V_ are the sensor horizontal and vertical dimensions, respectively, EFL is the effective focal length, and z_center_ is the aircraft-ground separation below the central pixel (Figure 3). All variables are measured in meters. First assuming a heading of 0° (i.e., North), each pixel was assigned a GPS location based on the frame size (1344 × 784 pixels):

(3) $$x_{\mathrm{pos},0\;}=\left(x_{\mathrm{center}}-\frac{H_{\mathrm{FoV}}}{2}:\frac{H_{\mathrm{FoV}}}{1344}{-\;1:x}_{\mathrm{center}}+\frac{H_{\mathrm{FoV}}}{2}\right),$$

(4) $$y_{\mathrm{pos},0}=\left(y_{\mathrm{center}}\;-\;\frac{V_{\mathrm{FoV}}}{2}:\frac{V_{\mathrm{FoV}}}{784}{\;-\;1:y}_{\mathrm{center}}+\frac{V_{\mathrm{FoV}}}{2}\right),$$

where x_center_ and y_center_ are the GPS coordinates at the central pixel. Note that this required the center pixel’s coordinates to be in the Universal Transverse Mercator (UTM) system, as H_FoV_ and V_FoV_ were measured in metres.

Next, a rotation matrix was applied to the coordinates using the GPS heading, θ, with the center pixel as the origin:

(5) $$\left[\begin{array}{cc} x_{\mathrm{pos}} & y_{\mathrm{pos}} \end{array}\right]=\left[\begin{array}{cc} \cos\theta & -\sin\theta \\ \sin\theta & \cos\theta \end{array}\right]\cdot \left[\begin{array}{c} {-x}_{\mathrm{pos},0}{+x}_{\mathrm{center}}\\ y_{\mathrm{pos},0\;}{-\;y}_{\mathrm{center}} \end{array}\right]+\left[\begin{array}{cc} x_{\mathrm{center}} & y_{\mathrm{center}} \end{array}\right]$$

**Figure 4:**
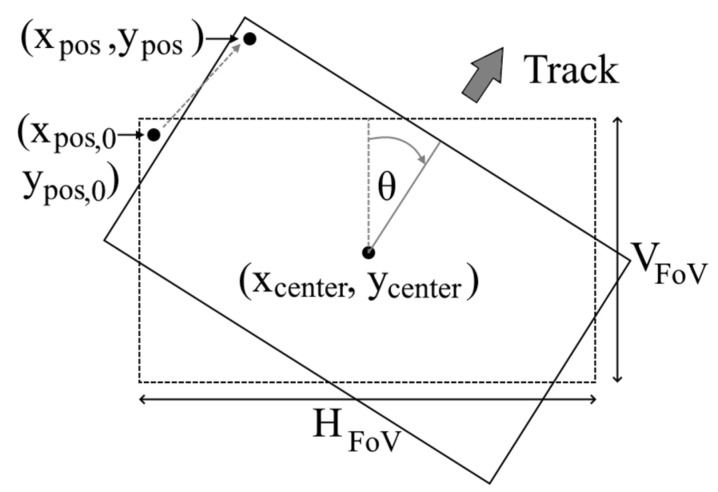
The initial pixel position assignment (x_pos,0_, y_pos,0_), shown in the dashed-line box. The post-rotation position (x_pos_, y_pos_) is shown in the solid-line box. H_FoV_ and V_FoV_ are the horizontal and vertical dimensions.

This gave a final matrix where each pixel (whose value was in raw counts) was assigned an initial estimated Easting (x_pos_) and Northing (y_pos_) location (Figure 4).

#### 3.1.3. Frame Registration

Once the data had initial geolocation estimates assigned, the process of building a geocorrected image using frame registration could begin. This was the most time-consuming process in the methodology presented here, as it is an iterative calculation, checking each pair of chronological frames in the IR video. Image registration is a process of automated data alignment, based on finding similarity in two images, and attempting to fit them together. This process is completed by centering a single image (the “fixed” image) and transforming a second image (the “moving” image), such that similarities between the two line up. The transform, T, is defined as:

(6) $$T=\left[\begin{array}{ccc} a & b & 0\\ c & d & 0\\ e & f & 1 \end{array}\right],$$

where the values of a to f are variable, depending on the type of transformation that is being allowed. Given the expected small variability in space and amplitude from one frame to the next, and the previous assumption of a reasonably flat, level, and straight flight line, the simplest transform, translation (T_translation_), was used for the processing:

(7) $$T_{\mathrm{translation}}=\left[\begin{array}{ccc} 1 & 0 & 0\\ 0 & 1 & 0\\ t_{x} & t_{y} & 1 \end{array}\right],$$

where t_x_ is the translation in the x direction and t_y_ is the translation in the y direction. Once T_transform_ was developed for a pair of frames, it was applied to the moving frame to arrive at the new, “registered”, coordinates:

(8) $$f_{r}\left(x,y\right){=f}_{f}\left(x,y\right){+f}_{f}\left(t_{x}x_{x}{+t}_{y}y_{x}{,\;t}_{x}x_{y}{+t}_{y}y_{y}\right),$$

where f_r_(x,y) is the moving frame’s registered coordinates, f_f_(x,y) is the fixed frame’s coordinates, x_x_ is the change in Eastings (in meters) of one x-value shift, x_y_ is the change in Northings (in meters) of one x-value shift, y_x_ is the change in Eastings (in meters) of one y-value shift, and y_y_ is the change in Northings (in meters) of one y-value shift. The last four of these variables are calculated from:

(9) $$x_{x}=y_{y}=d_{\mathrm{pixel}}\cos\theta ,$$

(10) $$x_{y}=y_{x}=d_{\mathrm{pixel}}\sin\theta ,$$

where d_pixel_ is the length of the (square) pixel.

**Figure 5:**
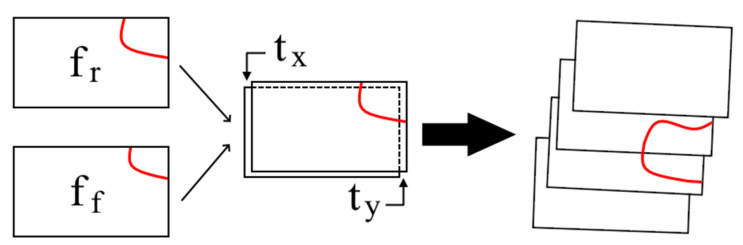
An outline of the general image registration process on an idealized feature (red line). Note that the spacing between the frames in the far right set have been exaggerated for clarity, and the middle pair of frames are more indicative of the usual spatial shift from frame to frame. f_r_ is the moving frame’s registered coordinates, f_f_ is the fixed frame’s coordinates, t_x_ is the translation in the x direction, and t_y_ is the translation in the y direction.

Applying this transformation, new coordinates for the moving frame were created, based on the fixed image’s coordinates. By iteratively moving through each frame, updating the moving and fixed frames in sequence, this process was repeated until all frames were registered (Figure 5).

#### 3.1.4. Gridding

**Figure 6:**
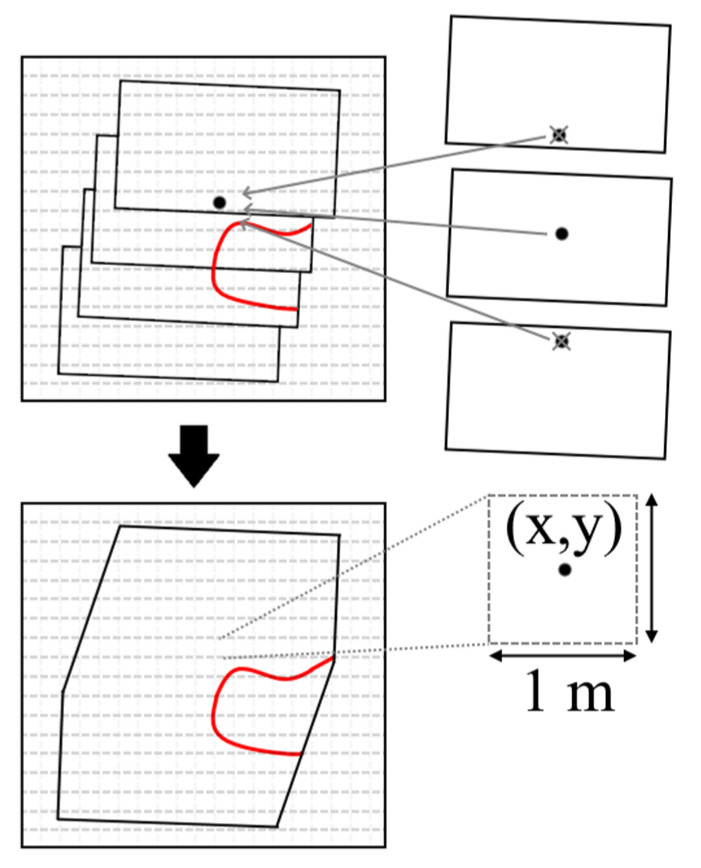
An exaggerated example of the gridding process on an idealized feature (red line). The dashed gray lines indicate the grid of cells. The left grids show the before and after representation of imagery during the gridding process. The three frames on the top right exemplify the process of determining what value should be in the final cell. Example shown for a 1 m pixel size.

With all frames aligned, a method to combine them into a single mosaic was required. To do this, a grid system, G_(x, y)_, was developed and data points from each frame were assigned to the grid in their respective grid cell locations. The cell size was chosen to be 1 m by 1 m, based on the average spacing between pixels, which was generally between 0.7 m and 0.8 m. This size ensured that no “holes” in the data would occur where no raw pixels existed. With many overlapping frames, most grid cells contained multiple data points, not all of which were the same value, and therefore a process to combine these was developed. A simple approach to this problem would be to take the mean of all data that falls within a cell. However, this causes an undesirable smoothing of the data. Due to the sharp edges of the fire features, this smoothing can mean a significant change in grid cell value. Instead, a modified minimum distance approach was used where the pixel that came from the FLIR frame whose center was closest to the grid cell of interest was found and its value used in the grid cell (Figure 6).

### 3.2. Georeferencing Process

**Figure 7:**
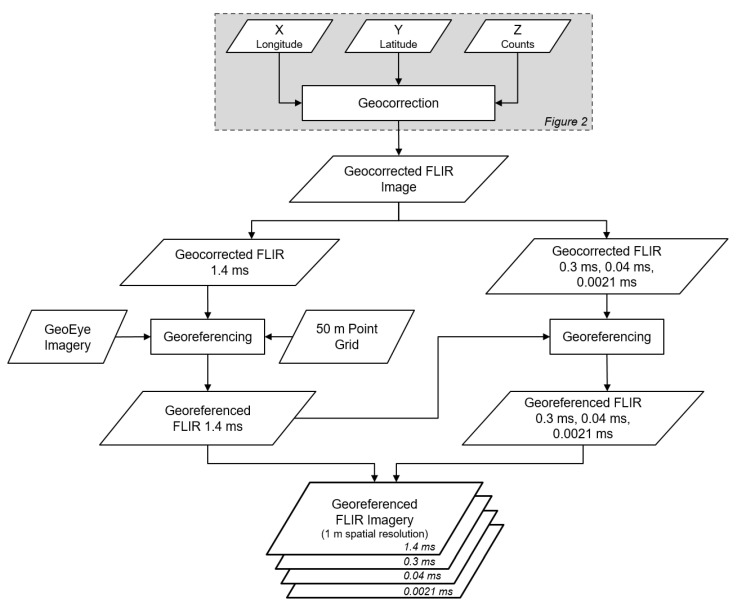
Workflow displaying the georeferencing methodology developed to reduce the geolocation error of the acquired FLIR data.

The geocorrection solution required further improvements to reduce the geolocation error (≤30 m) to a more acceptable threshold for comparison to mid-resolution satellite sensors (≤30–300 m). Figure 7 shows the georeferencing processing flow. The geocorrected FLIR data was georeferenced to GeoEye satellite imagery (2 m geolocation error) available in ArcGIS 10.6 (Environmental Systems Research Institute, Redlands, CA, USA). To reduce registration errors, the image with the longest integration time, 1.4 ms, was used as the baseline as it contained ambient temperature features. Firstly, the longitude (X dimension), latitude (Y dimension) information and the FLIR data in counts units (Z dimension) were exported from MATLAB and a geocorrected FLIR image was built in ENVI 5.5 (Exelis Visual Information Solutions, Boulder, CO, USA). Next, pseudo-ground control points (PGCPs) were registered throughout the FLIR image based on the GeoEye imagery in ENVI. To facilitate the selection of PGCPs, a point grid (50 m by 50 m) was created with GeoEye imagery over-layered in ArcMap. The first PGCP was selected over a recognizable area (e.g., riverbed), while the rest of the PGCPs were selected manually using the 50 m point grid at different distances from the initial point. Priority was given to PGCPs over recognizable areas (i.e., riverbed located around the image edges) and to points that did not cause significant image wrapping and showed less than 30 m error. Once a range of PGCPs was chosen, the FLIR imagery was georeferenced to the UTM Zone 16 N, WGS84 coordinate system at a pixel size of 1 m. The other integration times (i.e., 0.3 ms, 0.04 ms and 0.0021 ms) were subsequently georeferenced to the 1.4 ms integration time image for each of the flight lines.

### 3.3. Data Products

**Figure 8:**
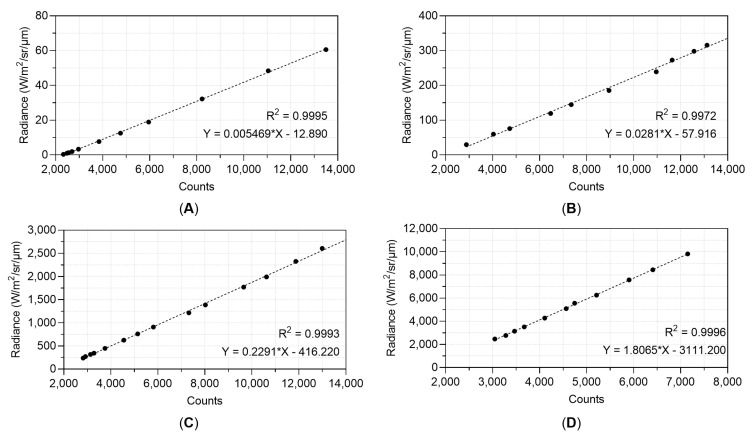
Relationship between counts recorded by the FLIR sensor and calculated radiance using the Planck function and a wavelength of 3.74 µm at integration times of (**A**) 1.4 ms, (**B**) 0.3 ms, (**C**) 0.04 ms, and (**D**) 0.0021 ms modified from [36]. The equations yielded by the trend line at each integration time were used to calculate radiance across each frame recorded by the sensor.

As mentioned in Section 2.2., calibration relationships between counts and temperatures were derived from the laboratory measurements. As temperatures are derived directly from the measured counts, calculation of the radiance produced at a specific wavelength from a specific temperature can be obtained by using the following version of the Planck function equation:

(11) $$L\left(\lambda ,T\right)=\frac{c_{1}}{\lambda^{5}(e^{\frac{c_{2}}{\lambda T}}-1)}\;$$

where λ is wavelength (µm) and in this case equal to the flame filter, 3.74 µm, T is brightness temperature (temperature when the emissivity = 1), L(λ,T) is spectral radiance in Wm^−2^ sr^−1^ µm^−1^, and *c*_1_ is the first radiation constant (value 1.191044024 × 10^−16^ Wm^2^), *c*_2_ is the second radiation constant (value 1.4387687 × 10^−2^ mK). As shown in Figure 8, linear calibration relationships were found between recorded counts and calculated radiance [36]. High confidence R^2^ values are reported at 0.9995 for 1.4 ms, 0.9972 for 0.3 ms, 0.9993 for 0.04 ms, and 0.9996 for the 0.0021 ms integration time. The equations yielded by the linear best fit were used to calculate radiance across each frame recorded by the sensor for each integration time.

Atmospheric correction was applied to the radiance values in the imagery in order to remove the influences of the atmosphere in accordance with convention for modelling the spectral radiance of a sub-pixel wildfire event [11]. The PcModWin 6.0.0.5 (Ontar Corporation, North Andover, MA, USA) software, a MODTRAN^®^ 6 [39] interface, was used to retrieve atmospheric variables for the correction process [40]. In this study, the standard MODTRAN parameters for sub-arctic summer atmospheric and rural aerosol models were used for the atmospheric compensation calculations.

To test the validity of the data, Fire Radiative Power Density (FRPD) was calculated using the MWIR method [11]. A threshold was applied to the data to remove the background and saturated pixel values. These background thresholds were based on data distribution at 10 Wm^−2^ FRPD for 1.4 ms, 60 Wm^−2^ FRPD for 0.3 ms, and 470 Wm^−2^ FRPD for the 0.04 ms integration time. Saturated pixels were defined as pixels with a count value above 13,500 because the detector’s behaviour beyond this value is highly non-linear and may either induce noise or provide false measurements. In this case, the saturation FRPD thresholds were 1098 Wm^−2^ for 1.4 ms, 5825 Wm^−2^ for 0.3 ms, and 48,500 Wm^−2^ for the 0.04 ms integration time. No saturated pixels were recorded at the shortest integration time 0.0021 ms. No fire pixels were recorded for FL-02 and FL-03 at the 0.0021 ms integration time during both nighttime (F-03) and daytime (F-04) flights.

## 4. Results

### 4.1. Geocorrection Assessment

**Figure 9:**
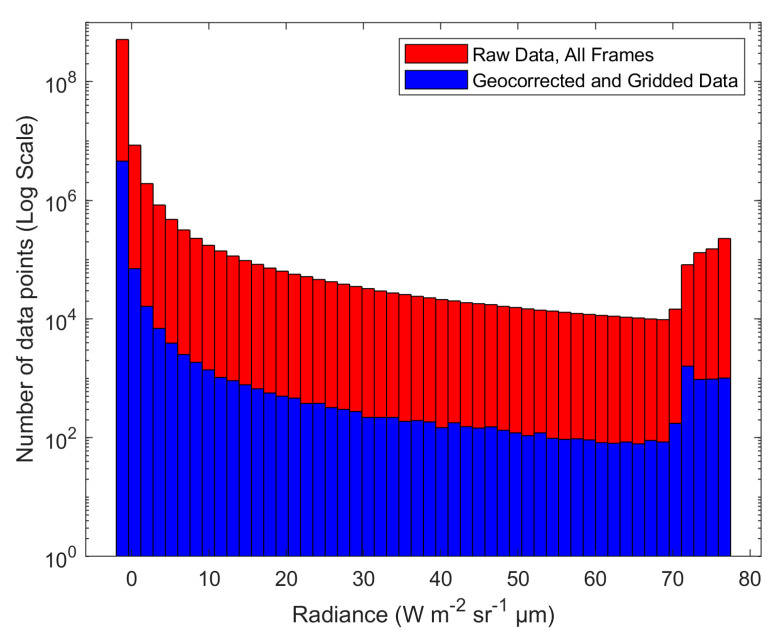
A histogram of the FLIR data in Radiance (Wm^−2^ sr^−1^ µm^−1^). The distribution of count values remains very similar through the geocorrection process, even though the amount of data has been greatly reduced.

To confirm the validity of the geocorrection process, we compared the distribution of data before and after it was applied to the daytime FL-04 dataset, acquired on F-04 with an integration time of 1.4 ms. In Figure 9 it can be seen that, while dramatically reducing the amount of data, the general distribution of radiance values remains highly similar after the geocorrection process. This is supported by the statistics of each dataset (Table 3), which indicate that the mean, median, minimum value, and maximum value have changed by less than 2%, while the standard deviation has changed by 7.14%. The skewness has only changed by 6.23%, however the kurtosis has deviated more, with an almost 13% change from the initial distribution to the geocorrected distribution. As the primary difference between the two distributions is in the higher radiance values (tail-end of the distribution), the larger kurtosis change compared to the other statistics is expected. Overall, the statistical changes between the two distributions are low, confirming that the geocorrection process has maintained the integrity of the raw FLIR data.

**Figure 10:**
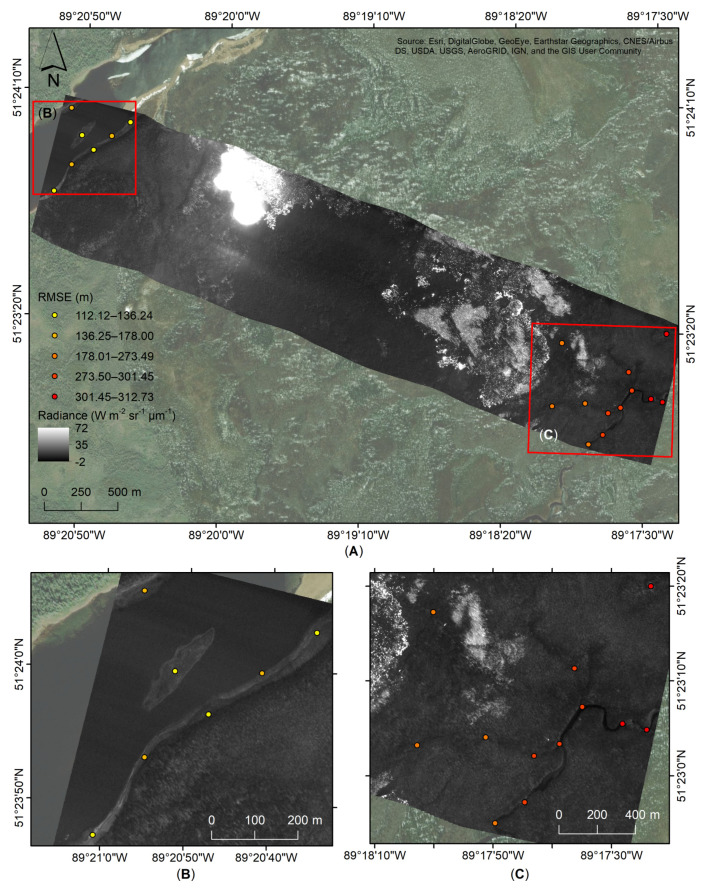
(**A**) An example of reported RMSE of the orthoimage (before the georeferencing process) in units of radiance (Wm^−2^ sr^−1^ µm^−1^) of FL-04, at an integration time of 1.4 ms, from F-04 acquired on 3 August 2017. Areas showing the misalignments and RMSE found following the geocorrection process for (**B**) the northwest and (**C**) southeast corners of the flight line.

An example of the raw imagery during daytime FL-04, acquired along F-04 at an integration of 1.4 ms, before georeferencing is shown in Figure 10. Misalignment issues can be observed at the edges of the imagery over the water bodies (Figure 10B,C). Based on 19 selected ground control points, a Root Mean Squared Error (RMSE) between 112.12 m and 312.73 m is reported. The mean RMSE found for FL-04, F-04, before the georeferencing step is at 233.99 m ± 73.28 m (based on 20 PGCPs). The northwest corner (Figure 10B) shows lower RMSE between 112.12 m and 178.01 m, while the southeast one (Figure 10C) shows higher RMSE between 178.01 m and 312.73 m.

### 4.2. Georeferencing Assessment

Table 4 shows the geolocation errors of the 1.4 ms integration time that was used as the baseline for the georeferencing process for the five processed flight lines. The total mean RMSE reported is 11.90 m ± 7.26 m with 6.96 m ± 5.21 m for Easting and 8.66 m ± 6.65 m for Northing. Absolute Easting error ranges between 0.30 m and 24.13 m, while Northing between 0.25 m and 25.43 m. The total minimum and maximum RMSE error are 0.87 m and 29.25 m, respectively. The Northing absolute median error is reported to be higher at 7.46 m, in comparison with Easting at 5.30 m, and a total RMSE error of 10.75 m.

**Figure 11:**
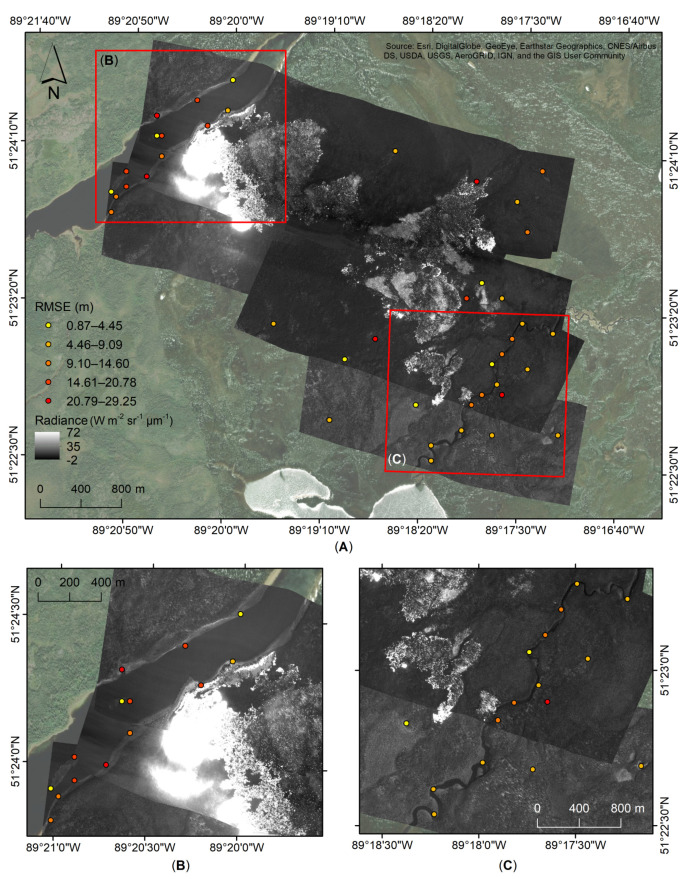
(**A**) Reported RMSE following the georeferencing process of the FLIR imagery in units of Radiance (Wm^−2^ sr^−1^ µm^−1^) acquired on 3 August 2017, F-04, at the 1.4 ms integration time. Areas showing RMSE following the georeferencing process for (**B**) the northwest and (**C**) southeast corners of the flight lines.

Figure 11 shows the reported RMSE, ranging from 0.87 m to 29.25 m, following the georeferencing process along Radiance (Wm^−2^ sr^−1^ µm^−1^) at an integration of 1.4 ms for the 3 August (F-04) flight lines. While, the northwest river shows variable RMSE with seven PGCPs showing above 14 m error (Figure 11B), a RSME below 14.60 m is reported along the southeast riverbed (Figure 11C).

To minimize geolocation errors between the 2 and 3 August datasets, the 2 August flight lines at 1.4 ms was georeferenced to the 3 August ones (Table 5). The mean total RMSE between the two datasets is 3.86 m, with a mean absolute Easting error of 2.38 m and 2.53 m for Northing. The total RMSE errors ranges between 0.17 m and 14.28 m with values between 0.01 m and 9.42 m for Easting and 0.04 m and 13.17 m for Northing. The total median RSME is 3.21 m at a standard deviation of 2.92 m.

Following the georeferencing process, the average RMSE errors is 15.11 m ± 3.54 m for the 2 August dataset and 13.53 m ± 2.60 m for 3 August (Table 6). Between 9 and 13 PGCPS were used in the georeferencing processing depending on the flight line. An RMSE between 8.73 m for FL-02 at 0.3 ms integration time, and 24.5 m for FL-05 for an integration time of 1.4 ms, can be observed. The 3 August dataset overall show lower RMSE errors between 8.83 m for FL-02 at an integration time of 1.4 ms, and 17.44 m for FL-05 at 0.04 ms. This is expected at the daytime flight show more landscape features that were used in the georeferencing process.

### 4.3. Data Products

**Figure 12:**
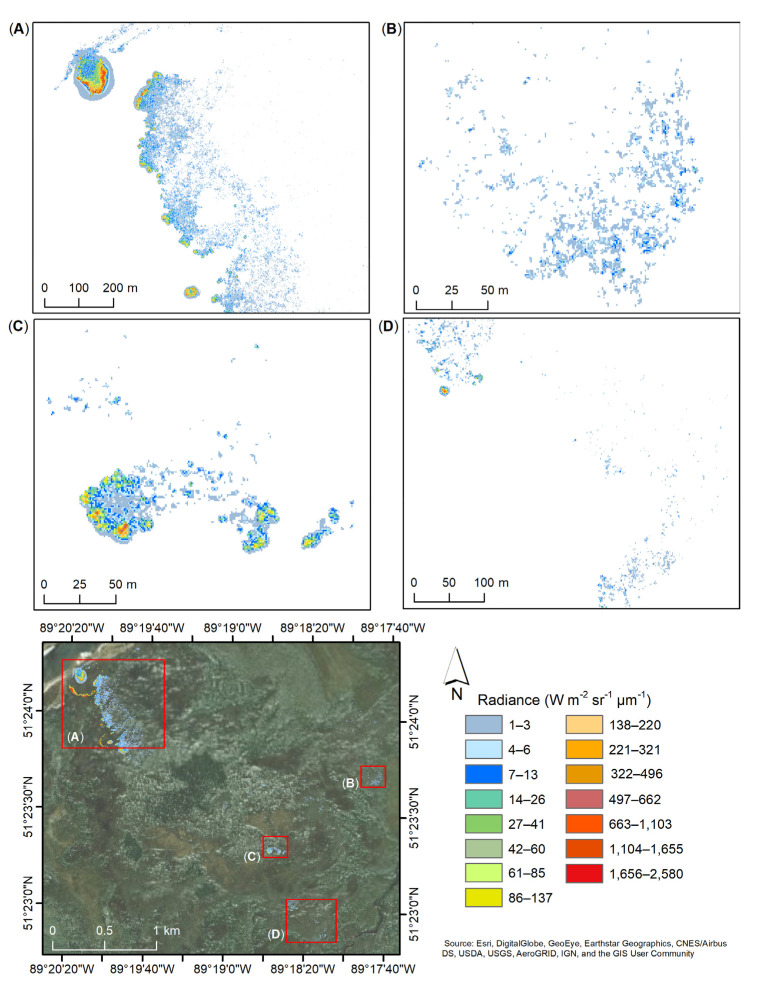
Radiance (Wm^−2^ sr^−1^ µm^−1^) captured during the nighttime flight, F-03, acquired on 2 August 2017, over four different area (**A**–**D**) of the SLK-37 site. Radiance of the 1.4 ms, 0.3 ms, 0.04 ms, and 0.0021 ms integration times superimposed based on the set thresholds (Section 3.3). Radiance below 1 Wm^−2^ sr^−1^ µm^−1^ (white) not displayed.

After thresholding (Section 3.3), all integration times’ datasets were superimposed upon each other creating a new combined dataset. Pixels with radiance below 1 Wm^−2^ sr^−1^ µm^−1^ were assigned as background and were not taken into consideration for the purpose of this analysis. Figure 12 shows the radiance (Wm^−2^ sr^−1^ µm^−1^) captured during the nighttime flight, F-03, on 2 August, where the presence of fire is detected over a few locations. In one particular area (Figure 12A), pixels with radiance values of up to 1765 Wm^−2^ sr^−1^ µm^−1^ are observed. A lower intensity radiance is shown in Figure 12C where fire pixels are present in different areas with a few pixels showing up to 662 Wm^−2^ sr^−1^ µm^−1^ and the majority of the fire pixels being between 6 and 26 Wm^−2^ sr^−1^ µm^−1^. In the southeast corner, more combustion pixel are observed at a radiance of up to 7 Wm^−2^ sr^−1^ µm^−1^ with a few pixels up to 13 Wm^−2^ sr^−1^ µm^−1^ (Figure 12B) and a small hotspot of up to 895 Wm^−2^ sr^−1^ µm^−1^ (Figure 12D).

**Figure 13:**
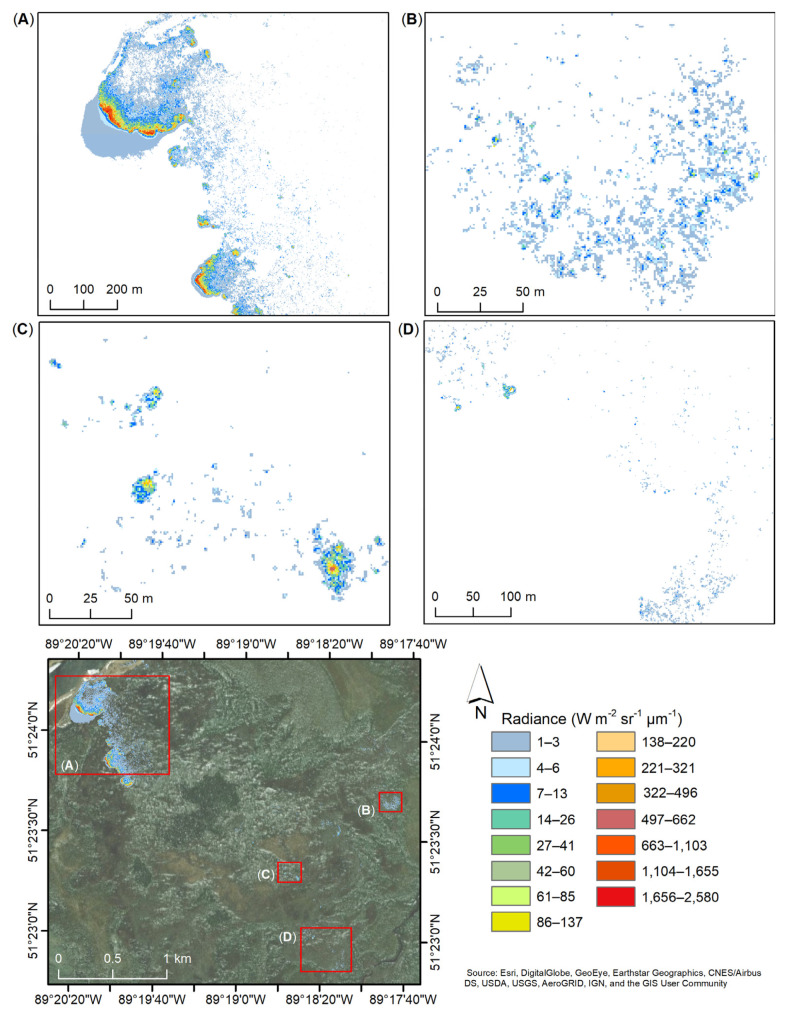
Radiance (Wm^−2^ sr^−1^ µm^−1^) captured during the nighttime flight, F-04, acquired on 3 August 2017, over four different area (**A**–**D**) of the SLK-37 site. Radiance of the 1.4 ms, 0.3 ms, 0.04 ms, and 0.0021 ms integration times superimposed based on the set thresholds (Section 3.3). Radiance below 1 Wm^−2^ sr^−1^ µm^−1^ (white) not displayed.

Similar trends are observed for the daytime flight, F-04, collected on 3 August (Figure 13). In the northwest side of the site (Figure 13A) the presence of fire extended and increased in intensity with radiance up to 2579 Wm^−2^ sr^−1^ µm^−1^ over two areas. These areas show marginally more thermal halo pattern generated by the hot soot within the smoke plume. The same mid-center area (Figure 13C), as that shown in Figure 12C, appears to have diminished in intensity, with radiance maximum of only 321 Wm^−2^ sr^−1^ µm^−1^, and increased within the area with a few pixels of up to 662 Wm^−2^ sr^−1^ µm^−1^. As recorded during the nighttime flight (FL-03), the southeast corner of the site shows radiance up to 13 Wm^−2^ sr^−1^ µm^−1^ (Figure 13B) and a small hotspot with a maximum radiance of 850 Wm^−2^ sr^−1^ µm^− 1^ in the north-west area (Figure 13D).

**Figure 14:**
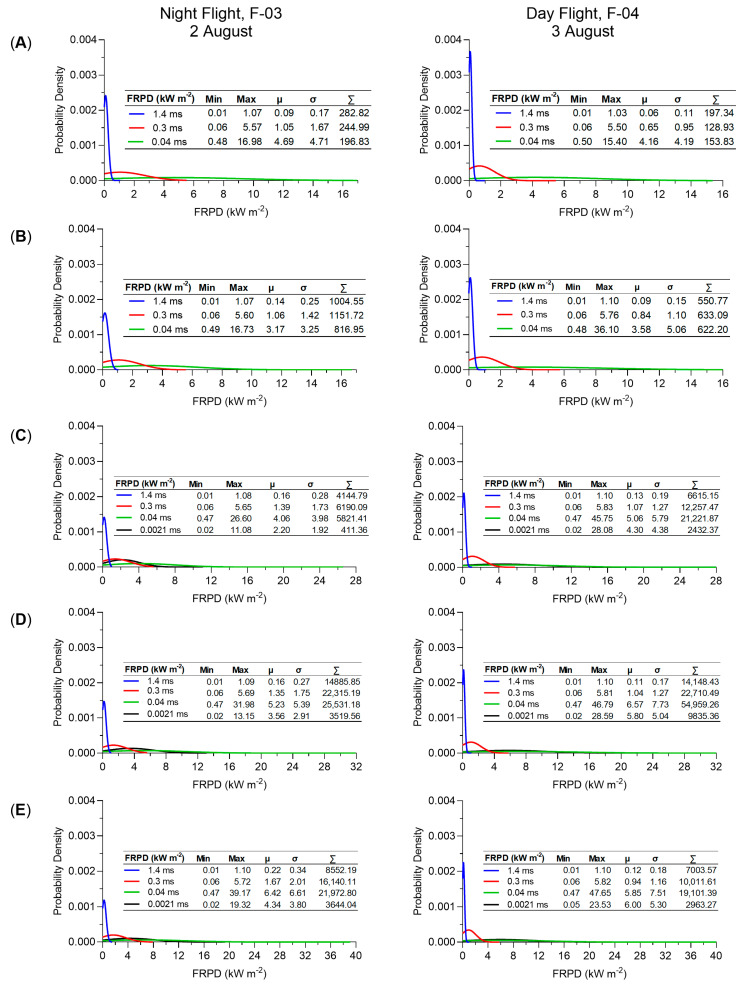
Probability density and statistics (minimum, maximum, mean, standard deviation and sum) of FRPD (kW^−2^) at the different integration times (i.e., 1.4 ms, 0.3 ms, 0.04 ms, and 0.0021 ms) for (**A**) FL-02, (**B**) FL-03, (**C**) FL-04, (**D**) FL-05, and (**E**) FL-06 acquired during night flight (F-03) and day flight (F-04) for the 2017 campaign.

Probability density and standard statistics (minimum, maximum, mean, standard deviation and sum) of FRPD (kWm^−2^) were computed for each flight line at the different integration times acquired during both nighttime and daytime flights (Figure 14). Given the dynamics of fire, FRPD for each flight line and integration time was calculated separately. 

For FL-02 (Figure 14A), higher FRPD are observed during the nighttime flight (F-03) with a maximum of 16.98 kWm^−2^ and a mean of 4.69 kWm^−2^ for the 0.04 ms, in comparison with the daytime flight (F-04) at a maximum of 15.40 kWm^−2^ and a mean of 4.16 kWm^−2^. Similar trends are seen for FL-03 (Figure 14B) where overall lower FRPD is recorded during the daytime. The FRPD recorded at the longer integration times decreased between nighttime flight (F-03) and daytime flight (F-04) from a sum of 1004.55 kWm^−2^ to 550.77 kWm^−2^ at 1.4 ms and from 1151.72 kWm^−2^ to 633.09 kWm^−2^ at 0.3 ms. Even though the FRPD at 0.04 ms is larger during the daytime flight (F-04), from a maximum of 16.73 kWm^−2^ to 36.10 kWm^−2^, the total area of FRPD decreased from 816.95 kWm^−2^ to 622.20 kWm^−2^ between night (F-03) and the day (F-04), respectively. This suggests that fire reduced in overall intensity but increased in FRPD over the area covered by FL-03. In contrast, higher FRPD were recorded over flight lines FL-04 to FL-06 (Figure 14C–E) in comparison to FL-02 and FL-03, but also during the daytime flight in comparison with the nighttime one. The highest FRPD is observed (Figure 14E) in the area covered by both flight lines FL-05 and FL-06 with a value of 47.65 kWm^−2^ for FL-06 at 0.04 ms. For FL-05 (Figure 14D), higher intensity FPRD is recorded during the day, from a maximum of 31.96 kWm^−2^ to 46.79 kWm^−2^ with a total FRPD also increasing from 25,531.18 kWm^−2^ to 54,959.26 kWm^−2^ from nighttime flight (F-03) to daytime flight (F-04).

**Figure 15:**
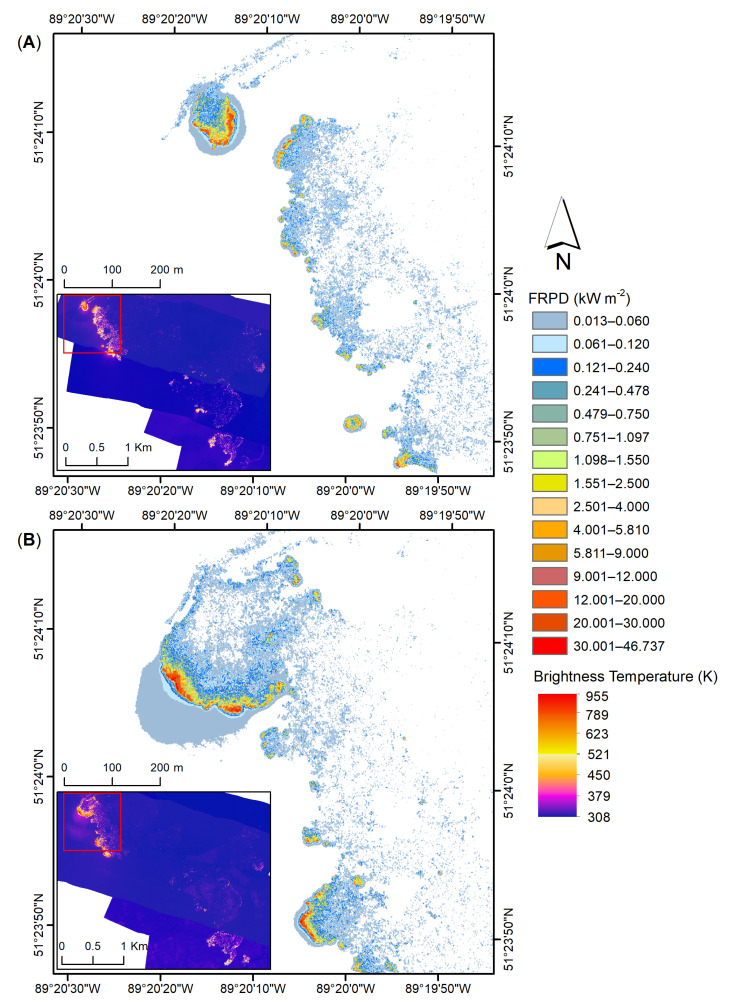
Example of FRPD (kWm^−2^) captured over FL-05 during both (**A**) nighttime flight, FL-03, on 2 August, and, (**B**) daytime flight, F-04, on 3 August, during the 2017 campaign over the SLK-37 site. Calculated FRDP of the 1.4 ms, 0.3 ms, 0.04 ms, and 0.0021 ms integration times superimposed based on the set thresholds (Section 3.3). Overall inset area shown in brightness temperature (K) as a reference.

Figure 15 showcases an example of the highest FRPD recorded over FL-05 during both nighttime and daytime flights. The distributions of FRPD for the night (Figure 15A) and day (Figure 15B) flights clearly demonstrate the bias toward higher FRPD values during the day (i.e., increased flaming combustion) and lower FRPD during the night (i.e., increased smouldering combustion), which is consistent with diurnal trends in fire behaviour. As observed in the radiance (Figure 13A), the day flight FRPD shows marginally more halo generated by the soot in the smoke plume.

## 5. Discussion

Our study provides a novel geocorrection methodology for a case where GPS and IMU airborne equipment malfunctions occurred during a 2017 airborne wildfire campaign collecting FLIR MWIR images. This solution required developing an approach to assign GPS coordinates to FLIR video imagery, followed by combining the data through image registration. This process alone, while effective, required an additional georeferencing method to reduce the RMSE of the positional accuracy. Using this approach, the RMSE was reduced from a baseline error of up to 300 m (Figure 10) to an average of 15.11 m for the nighttime and 13.53 m for the daytime datasets (Figure 11, Table 6). By comparison, under optimal equipment conditions, a similar method for the geocorrection of ATIR FireMapper 2.0 airborne infrared imagery, acquired to estimate fire spread rates in southern California, shows a geocorrection RMSE of 0.33 pixels (4.13 m) [24]. In addition, Ref. [41] reports up to 2 m along-track error when using a georeferencing gridding in combination with associated GPS and IMU information for processing of airborne Specim AISA Eagle and Hawk hyperspectral data. Using a frame center matching technique, Ref. [42] was able to reduce misregistration errors of multi-temporal ADAR 5500 airborne imagery. The study found RSME errors of up to 8.30 m when using automatic ground control points during the georeferencing process in combination with introducing a DEM during the geocorrection process. Using the same frame registration method, Ref. [43] found an average 2.1 pixels (0.32 m) geolocation error, which can increase to 4.4 pixels (0.67 m) for moderate terrain (95–105 m elevation, 10–20 m slope) and up to 16.2 pixels (2.47 m) for extreme terrain (145–155 m elevation, 20–30 m slope), after orthorectification of ADS40 imagery. These methods show lower errors in comparison with our study due to the use of DEM, LIDAR and GPS/IMU data in the geocorrection process.

Our study also relied on GeoEye imagery available through the ArcGIS software, which has a reported error of 2 m [44]. Although not necessarily optimal by comparison, our approach was successful in recovering what otherwise may have been unusable data. Given the initial purpose of comparing this dataset to satellite imagery (30 m resolution) we considered the overall below 30 m RMSE error (Figure 11, Table 4, Table 5 and Table 6) to be acceptable when taking into consideration the extra cost and processing time involved in reducing further the geolocation error.

The distribution of raw FLIR data as compared to the gridded and geocorrected dataset (Figure 9) and associated statistics (Table 3) are key to showing that this method is not biasing the data. The gridding process, which effectively distills 99% of the frame data to arrive at the final, single dataset, should not be averaged. As described in Section 3.1.4., the sharp peaks of the fire, represented by the tail ends of the distributions, would be affected by an averaging process, smoothing out the results. Keeping the tail of the distribution is important to maintain, as this is where most of the information in the data products (e.g., FRPD) is held. Even without averaging, it is clear that the largest difference between the two distributions is in the tail, indicated by the change in the kurtosis (12.98%) values. This change, while still overall small, is due to the sampling process during gridding. The peaks of the fires were generally one or two pixels large, and as such, they were more likely to be affected by the cell size being slightly larger than the raw pixel size. This caused the peak values (the right side of the distribution tail) to be disproportionately lost during the gridding’s subsampling process, thus leading to a slight increase in representation on the left side of the tail (the more numerous fire values surrounding the peaks). However, the shift is small (radiance of high 80 s to low 70 s) and contained to the tail of the distribution. This is supported by the statistics, particularly the change in mean (1.70%), median (0%), and standard deviation (7.14%) being low. For these reasons, we are confident that the gridding process did not bias or alter the raw FLIR data by a significant amount.

A primary limitation to this method is that the initial georeferencing process assumes the sensor was pointed nadir in the aircraft. Unfortunately, without any IMU information this will always be a necessary assumption (unless of course the camera is mounted at an angle on the airborne platform). This assumption is the key reason that the georeferencing step was developed in our methodology. Even with a level flight path, small deviations (primarily variations in the instantaneous roll and pitch angles) will add up over the course of the flight line, leading to an accumulation of positional errors. This can be clearly seen in Figure 10, where the eastern side of the image has a greater positional inaccuracy as compared to the western side where the image registration process began. The initial frame of data will also influence this “drift” of error. We use the GPS data and ground elevation to determine the coordinates for all pixels in this initial frame. However, all subsequent frames are registered against this, and as such, any error in the locations due to a small pitch or roll during this initial frame will be carried through all other frames. The georeferencing step greatly alleviates these overall errors, as can be seen in the final positional error analysis (Figure 11, Table 6). An area for future investigation would be to determine if there is a method of using any GPS information (in this case, the 1 Hz real-time processed locations) during the image registration process to develop a combined solution. This would likely assist in reducing the drift error, thus requiring less adjustment during the georeferencing process.

The decision to limit the image registration process to only the translation transform came about after experimentation and analysis was performed with the other transformation options, namely rotation, scaling, and shearing factors. Due to the 7.5 Hz collection rate for an individual integration time, combined with the straight and level flying during the survey, little would change between most frames. Therefore, even when given the additional freedom of transforms, the translation parameters would dominate.

Based on the MWIR radiance method [11], our study shows FRPD values up to 39.17 kWm^−2^ (Figure 14 and Figure 15A) during the nighttime and up to 47.65 kWm^−2^ (Figure 14 and Figure 15B) for data acquired during daytime. This is consistent with diurnal trends in fire behaviour [45] where increased flaming combustion is observed during the day and increased smouldering combustion during the night. Even though our study found an FRPD of up to 48 kWm^−2^, based on the probability density results (Figure 14) most pixels show values up to 6 kWm^−2^ with a few showing the fire front (Figure 15) between 9 and 47 kWm^−2^. Though occupying a far larger combustion zone, our FRPD and temperature range observations correspond with measurements taken in study [11] that describes the MWIR method. Over a 50-min fire, the study found a peak of 7 kW with most reported values between 5 and 2 kW. Furthermore, at temperatures of 900–950 K they report FRPD values (referred to as FRP) of up to approximately 50–60 kWm^−2^. In comparison, our data shows temperatures up to 955 K (Figure 15) for a FRPD of up to 47 kWm^−2^. Other studies across the literature reported similar fire intensity data ranges. Using an AGEMA 550 thermal camera, Ref. [9] found an FRP of up to 6 kW for smouldering fire and between 15 and 35 kW FRP for flaming fire for different small-scale fire scenarios (1.0–1.5 cm pixel resolution) collected at an off nadir angle. In the same study, fire intensity was investigated over an open burn plot acquired with the same AGEMA 550 thermal camera attached to a helicopter. They found an FRPD (referred to as FRF) between 12 and 14 KWm^−2^ of a 500 s controlled burn fire. Another study reports a radiative fire intensity between 50 and 250 kWm^−1^ when using the AGEMA 550 and FLIR SC6703 sensors [31]. Based on the current literature, our FRPD values are within expected/compared values in other studies.

The initial goal of this survey was to compare the airborne infrared imagery to coincident satellite imagery to complete a multiscale analysis, specifically the Sentinel-3 SLSTR, which suffered a protective shutdown during the flight campaign [46]. Based on the Mission Status report [46], the satellite experienced a computer double bit-error and no data was acquired between 31 July and 6 August 2017. This malfunction happened as it passed through the South Atlantic Anomaly, an area of increased radiation due to increased proximity to Earth’s surface. The relevance of geocoding airborne wildfire imagery extends beyond its use in evaluating space-borne retrievals. Once corrected, this imagery provides significantly more value in terms of characterizing wildfire fire radiative power [9], rate of spread [47] and fire-line intensity [31] providing the unique capacity to study a naturally occurring wildfire without the need for ground sampling. Furthermore, our approach is valuable in experimental situations where the availability of precise positional data is limited (e.g., [9]) due to budget and or aircraft type (e.g., short term contract and/or UAV).

While the method and results described here are specific to the 2017 field campaign data shown, in essence, this method solves the georeferencing step in any airborne campaign where no high-frequency, high-accuracy IMU data is collected. As such, with minimal effort, most of the process can be used in other situations and contexts. To facilitate this, open-source MATLAB code of the image registration and gridding processes has been released [38]. Some effort is required by the user to prepare their data to fit this methodology, most notably setting the initial position estimations for the first frame in the dataset. However, the process itself requires basic expertise in MATLAB or image processing, and an example dataset (several frames from the wildfire campaign presented here) is given to guide a user through the requirements.

## 6. Conclusions

To validate satellite products, an airborne campaign was carried out to collect thermal imaging of wildland fires near Pickle Lake, Northern Ontario, in August 2017. For this campaign, multiple BOB fires were imaged using an on-board MWIR broadband FLIR SC8303 sensor equipped with a flame filter (3.74 µm) at four different integration times (i.e., 1.4 ms, 0.3 ms, 0.04 ms, and 0.002 ms). Unfortunately, there were two separate points of hardware failure during the data acquisition: the sensor never received GPS timing information and the aircraft’s IMU failed to record. An alternative geolocation method was then developed to address these two major issues. The FLIR frames were aligned with a backup GPS-tagged camera and assigned geolocations, before being combined into full orthoimages through an image-registration process that did not require IMU information. The orthoimages were then further georeferenced using a developed grid system to improve the geolocation error. This second step was necessary to allow for an effective comparison with mid-resolution satellite sensors (≤30–300 m). Using our approach, the geolocation RMSE error was reduced from up to 300 m to 15.11 m for the nighttime data set and to 13.53 m for the daytime dataset. Our study also shows that the computed FRPD estimations, derived from the geocorrected FLIR data, are within expected values across the literature. Overall, the process led to an effective geocorrection of the infrared data, which has since been used for various analyses, such as fire radiative power, to inform wildfire scientists.

The methodology here can be easily adapted and applied to any wildfire campaign where no IMU or GPS information is available, whether due to a lack of hardware or hardware issues like those in the wildfire campaign presented here. It is a simple and effective basis for orthoimage creation that will keep sharp edges present at wildfire peaks, while being geographically accurate for comparison to associated satellite imagery, or other airborne data.

## Figures and Tables

**Table 1:** Summary of presented FLIR data acquired over the Sioux Lookout (SLK-37) site, located near Pickle Lake, Northern Ontario, Canada, during the 2017 campaign. Average altitude and ground speed were extracted from the aircraft’s avionics GPS system.

Flt No.	Date	Time Period	No. of Lines	Start Time (EST)	End Time (EST)	Altitude (m MSL)	Ground Speed (Knots)
F-03	August 2 ^a^	Night	16 ^b^	22:20	23:37	2888.2	92.5
F-04	August 3	Day	16 ^b^	10:26	11:41	2915.1	93.9

^a^ UTC = Local (EST) +5. ^b^ Eight flight lines acquired twice.

**Table 2:** Summary of key characteristics of the SC8303 FLIR MWIR sensor.

Characteristic	Value
Spectral Range (µm)	3.0–5.0
Detector Pitch (µm)	14
Frame Rate (Hz)	Up to 125
Resolution (pixels)	1344 × 784
Temperature Accuracy	±2 or 2%
Standard Temperature Range (°C)	−20 to +350
Operating Temperature Range (°C)	−40 to 50
Weight without lens (kg)	4.5

**Table 3:** Statistics of all frames of raw data, and the geocorrected, gridded data. Despite the reduction of data points to arrive at the gridded dataset, the data has not been greatly altered, indicated by the similar statistics of the two datasets. Note that all values, other than the total number of data points, skewness, and kurtosis, are in radiance (Wm^−2^ sr^−1^ µm).

Variable	Raw, All Frames	Geocorrected, Gridded	Change (%)
Total Data Points	527,901,696	4,701,690	99.11
Mean	−1.18	−1.20	1.70
Median	−1.44	−1.44	0.00
Standard Deviation	3.08	2.86	7.14
Minimum	−1.66	−1.64	1.21
Maximum	77.39	77.05	0.44
Skewness	21.18	22.50	6.23
Kurtosis	489.75	553.33	12.98

**Table 4:** Reported absolute (Abs.) georeferencing errors for the FLIR imagery acquired on 3 August 2017, at the longest integration time of 1.4 ms of the five processed flight lines. Results reported over 51 pseudo-ground control points (PGCPs).

Variable	Abs. Easting (m)	Abs. Northing (m)	Total RMSE (m)
Mean	6.96	8.66	11.90
Minimum	0.30	0.25	0.87
Maximum	24.13	25.43	29.25
Median	5.30	7.46	10.75
Standard deviation	5.21	6.65	7.26

**Table 5:** Reported absolute (Abs.) georeferencing errors for the data acquired on 2 August (F-03) at the longest integration time of 1.4 ms to the baseline imagery collected at an integration of 1.4 ms on 3 August (F-04) during the 2017 campaign over the SLK-37 site.

Variable	Abs. Easting (m)	Abs. Northing (m)	Total RMSE (m)
Mean	2.38	2.53	3.86
Minimum	0.01	0.04	0.17
Maximum	9.42	13.17	14.28
Median	2.02	1.55	3.21
Standard deviation	1.96	2.76	2.92

**Table 6:** Reported total RMSE (m) of pseudo-ground control points (PGCPs) for data acquired during night flight (F-03, 2 August) and day flight (F-04, 3 August) during the 2017 campaign over the SLK-37 site.

Flight Line		Night Flight (August 2)		Day Flight (August 3)	
	IT (ms)	PGCPs	RMSE (m)	PGCPs	RMSE (m)
FL-02	1.4	9	10.85	10	8.83
	0.3	9	8.73	10	8.83
	0.04	9	9.67	9	9.71
FL-03	1.4	9	16.22	9	14.28
	0.3	10	14.54	9	14.28
	0.04	10	14.54	9	14.93
FL-04	1.4	9	17.32	13	11.76
	0.3	10	12.46	13	11.76
	0.04	9	13.36	12	12.58
	0.0021	9	13.36	12	12.39
FL-05	1.4	9	24.50	10	16.65
	0.3	10	16.99	10	16.65
	0.04	9	18.21	12	17.44
	0.0021	9	18.21	12	17.33
FL-06	1.4	10	17.33	10	13.84
	0.3	10	14.57	10	13.84
	0.04	9	15.58	12	14.64
	0.0021	9	15.58	12	14.62

## Data Availability

Not applicable.
